# Supplementary material for: Inflammatory factors and risk of lung adenocarcinoma: a Mendelian randomization study mediated by blood metabolites
Source: Front Endocrinol (Lausanne). 2024 Aug 27;15:1446863. doi: 10.3389/fendo.2024.1446863 (PMC11384989; doi:10.3389/fendo.2024.1446863)
Supplement: Supplementary file 1 [file DataSheet1.zip › supplementary files/S2/GCST90200241/forest.pdf]

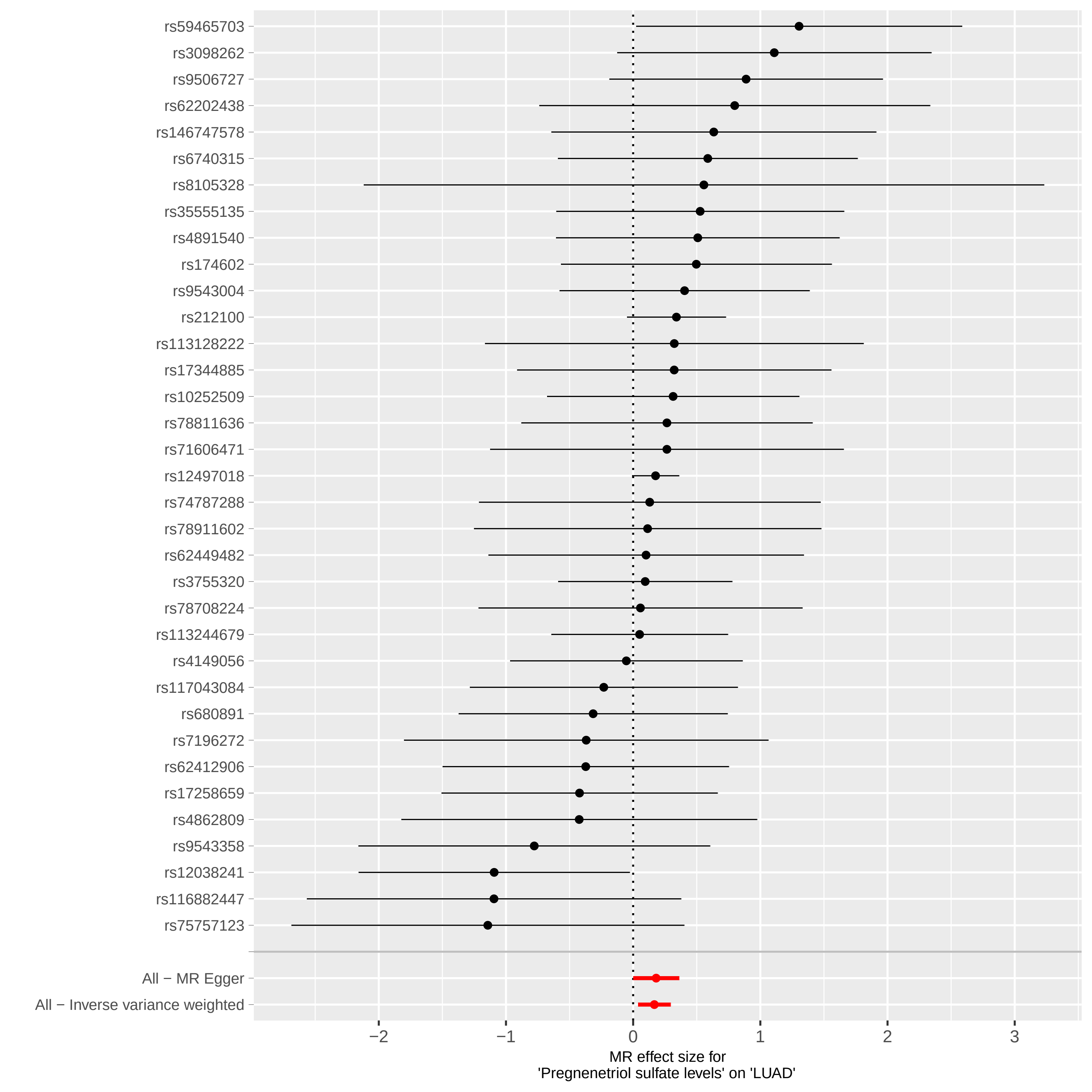
rs59465703
rs3098262
rs9506727
rs62202438
rs146747578
rs6740315
rs8105328
rs35555135
rs4891540
rs174602
rs9543004
rs212100
rs113128222
rs17344885
rs10252509
rs78811636
rs71606471
rs12497018
rs74787288
rs78911602
rs62449482
rs3755320
rs78708224
rs113244679
rs4149056
rs117043084
rs680891
rs7196272
rs62412906
rs17258659
rs4862809
rs9543358
rs12038241
rs116882447
rs75757123
All − MR Egger
All − Inverse variance weighted
−2
−1
0
1
2
3
MR effect size for
'Pregnenetriol sulfate levels' on 'LUAD'
